# Exploring relationships among multi-disciplinary assessments for knee joint health in service members with traumatic unilateral lower limb loss: a two-year longitudinal investigation

**DOI:** 10.1038/s41598-023-48662-9

**Published:** 2023-12-01

**Authors:** Joseph G. Wasser, Brad D. Hendershot, Julian C. Acasio, Lauren D. Dodd, Rebecca L. Krupenevich, Alison L. Pruziner, Ross H. Miller, Stephen M. Goldman, Michael S. Valerio, Lien T. Senchak, Mark D. Murphey, David A. Heltzel, Michael G. Fazio, Christopher L. Dearth, Nelson A. Hager

**Affiliations:** 1 Department of Rehabilitation, Walter Reed National Military Medical Center, Bethesda, MD USA; 2 Henry M. Jackson Foundation for the Advancement of Military Medicine, Inc., Bethesda, MD USA; 3 Department of Physical Medicine and Rehabilitation, Uniformed Services University of the Health Sciences, Bethesda, MD USA; 4 Research and Surveillance Section, Extremity Trauma and Amputation Center of Excellence, Research and Engineering Directorate, Defense Health Agency, Falls Church, VA USA; 5 Department of Kinesiology, University of Maryland, College Park, MD USA; 6 Department of Surgery, Uniformed Services University of the Health Sciences, Bethesda, MD USA; 7 Department of Diagnostic Radiology, Walter Reed National Military Medical Center, Bethesda, MD USA

**Keywords:** Trauma, Biomedical engineering, Risk factors

## Abstract

Motivated by the complex and multifactorial etiologies of osteoarthritis, here we use a comprehensive approach evaluating knee joint health after unilateral lower limb loss. Thirty-eight male Service members with traumatic, unilateral lower limb loss (mean age = 38 yr) participated in a prospective, two-year longitudinal study comprehensively evaluating contralateral knee joint health (i.e., clinical imaging, gait biomechanics, physiological biomarkers, and patient-reported outcomes); seventeen subsequently returned for a two-year follow-up visit. For this subset with baseline and follow-up data, outcomes were compared between timepoints, and associations evaluated between values at baseline with two-year changes in tri-compartmental joint space. Upon follow-up, knee joint health worsened, particularly among seven Service members who presented at baseline with no joint degeneration (KL = 0) but returned with evidence of degeneration (KL ≥ 1). Joint space narrowing was associated with greater patellar tilt (r[12] = 0.71, *p* = 0.01), external knee adduction moment (r[13] = 0.64, *p* = 0.02), knee adduction moment impulse (r[13] = 0.61, *p* = 0.03), and CTX-1 concentration (r[11] = 0.83, *p* = 0.001), as well as lesser KOOS_Sport_ and VR-36_General Health_ (r[16] = − 0.69, *p* = 0.01 and r[16] = − 0.69, *p* = 0.01, respectively). This longitudinal, multi-disciplinary investigation highlights the importance of a comprehensive approach to evaluate the fast-progressing onset of knee osteoarthritis, particularly among relatively young Service members with lower limb loss.

## Introduction

Knee osteoarthritis (OA) is commonly diagnosed through radiographic indicators, clinical examinations, and patient-reported symptoms^1^. Upon radiographic evaluation, formation of osteophytes and diminished joint space indicate deterioration of the knee joint, leading to OA pathology^2^. Furthermore, increased pain severity and functional limitations are clinically meaningful manifestations associated with symptomatic knee OA^3,4^. While degenerative OA has traditionally been associated with advancing age, earlier onset is more prevalent among younger adults after limb trauma^5,6^; for example, higher prevalence rates in younger Service members (SM) with (unilateral) lower limb loss compared to the general population (28% versus 12%)^5,7,8^. Earlier onset of knee OA can substantially reduce long-term functionality and quality of life^7^; thus, better understanding risk factors for longitudinal changes in knee joint health among SM with lower limb loss is critical for military medicine.

In the non-limb loss population, cross-sectional and longitudinal studies of knee OA have identified/monitored several factors associated with knee OA onset and progression^9,10^, highlighting the multifactorial nature of the pathology and motivating the need for a comprehensive evaluation. Biomechanical factors play an important role in joint health, and altered joint loading has been associated with both the onset and progression of knee OA^11^. Altered joint loading is of particular concern for persons with (unilateral) lower limb loss, who typically walk and perform activities with a preference for the contralateral (intact) limb^12,13^, thus theorized to increase risk for knee joint degeneration due to repetitive exposures to larger and/or prolonged mechanical loads. Considering that such repeated exposures to abnormal loading may not result in near-term structural damage or symptoms, physiological biomarkers can provide additional insights into the underlying molecular and cellular indicators of joint health, perhaps even facilitating early(ier) identification of disease onset^14^. For example, elevated concentrations of markers indicative of inflammation and/or osteochondral remodeling are present in both serum and synovial fluids with OA^15,16^. Degraded cartilage material properties, modulated by the composition of the extracellular matrix, have also been related to synovial inflammation and increases in cartilage strain for a given load^17^. Yet, despite the high prevalence (and seemingly early onset) of knee OA in persons with lower limb loss, coupled with the complex interactions among the biomechanical environment with both joint structure and physiology, to date no studies have utilized a comprehensive approach to explore knee joint health after limb loss.

This study aimed to identify longitudinal changes in associative risk factors for OA, from a comprehensive assessment for knee joint health in SM with unilateral lower limb loss. We hypothesized that decreases in medial tibiofemoral and patellofemoral joint spaces would be positively associated with increased knee joint kinetics and elevated concentrations of physiological markers of bone and cartilage degradation and remodeling, and inflammation. Moreover, we predicted that radioanatomic measures such as bisect offset, patellar tilt, and tibiofemoral joint angle would be associated with greater medial patellofemoral joint space narrowing. Diminished knee joint health would also be associated with poorer patient-reported outcomes (i.e., greater pain, reduced function or quality of life). Better understanding longitudinal relationships among morphological, biomechanical, and physiological factors associated with knee OA after lower limb loss is a necessary first step toward mitigating the deleterious effects of diminishing knee joint health and maximizing long-term outcomes for SM with lower limb loss.

## Methods

### Participants

**Figure 1:**
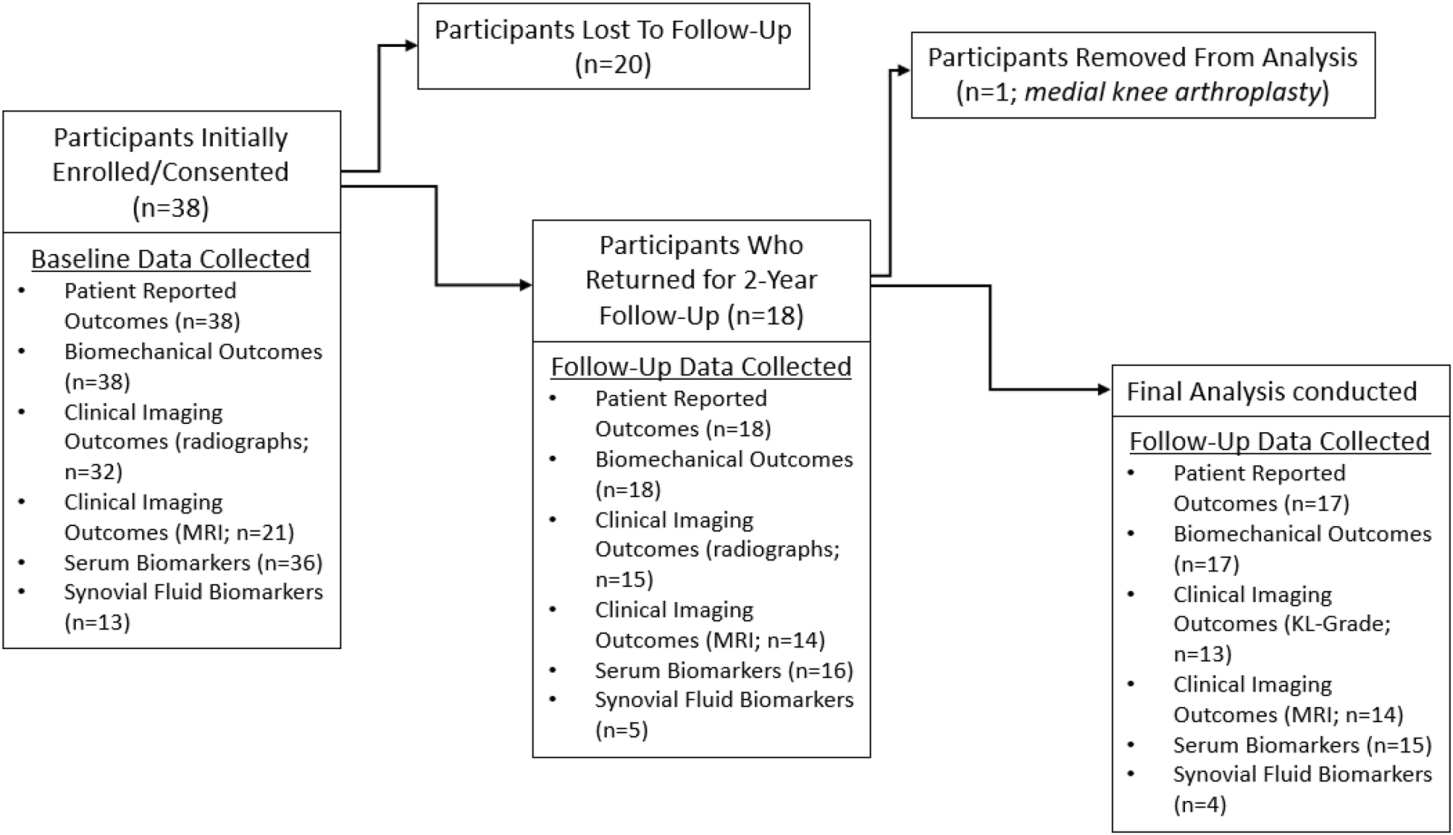
Study flow diagram to clarify participation, outcomes, and loss to follow-up.

Thirty-eight male SM with unilateral lower limb loss (23 transfemoral and 15 transtibial; mean ± standard deviation age: 37.9 ± 6.5 yr; stature: 1.78 ± 0.05 m; body mass: 89.2 ± 18.4 kg; time since injury: 114.4 ± 70.9 months) were enrolled into a prospective, two-year longitudinal study utilizing a comprehensive evaluation (i.e., clinical imaging, gait biomechanics, physiological biomarkers, and patient-reported outcomes) for identifying the onset, progression, and overall impact of knee OA on functionality and quality of life; eighteen (9 transtibial and 9 transfemoral) SM returned for the two-year follow-up. One participant was removed from subsequent analyses due to receiving a medial knee arthroplasty between baseline and follow-up; thus, seventeen SM with unilateral lower limb loss (9 transtibial and 8 transfemoral) were included (Fig. 1). The Walter Reed National Military Medical Center Institutional Review Board approved all experimental procedures (protocol #500081), performed in accordance with all relevant guidelines and regulations, and written informed consent was obtained from all participants prior to testing.

### Clinical imaging

Standard, bilateral weight-bearing anterior–posterior and sunrise-view radiographs of the intact knee were obtained for 15 SM at baseline and follow-up (n = 2 were not medically cleared). Imaging outcomes include clinical grading of knee joint health via Kellgren-Lawrence (KL) classifications and the modified Outerbridge (OC) grading scale (for medial tibiofemoral compartment, lateral tibiofemoral compartment, and patellofemoral joint), measurement of medial and lateral tibiofemoral, medial and lateral patellofemoral joint space narrowing, and radioanatomic positions of the knee and patella. Radiographic OA within the intact knee was considered at KL grade ≥ 2. Patellar tilt angle^18^, sulcus angle^19^, bisect offset^18^, femoral-tibial angle^20^, knee rotation^21^, and Insall-Salvati ratio^22^ were also measured, as described previously.

### Gait biomechanics

All SM at baseline (n = 17) and two-year follow-up (n = 17) completed a gait evaluation, walking at a self-selected pace along a 15 m walkway. Full-body kinematics were obtained using an 18-camera motion capture system (Qualisys, Göteborg, SE) to track (120 Hz) the positions of 62 reflective markers. Markers were placed on the head (× 4), C7 and T10 spinal processes, sternal notch, xiphoid, bilaterally across the acromion, humeri, lateral elbows, forearm, radii, ulnae, dorsal hand, posterior and anterior superior iliac spines, calcanei, hallux, and the 2nd and 5th metatarsals. Cluster-based tracking markers were also placed bilaterally on each thigh and shank (4 markers per cluster). Twelve additional calibration-only markers were placed bilaterally on the medial epicondyle of the elbow, lateral and medial epicondyles of the knee and ankle maleoli, and the medial aspect of the 1st metatarsal. Bilateral ground reaction forces were simultaneously sampled (1200 Hz) from six force platforms (AMTI, Watertown, MA, USA) embedded within the walkway. Marker trajectories and ground reaction forces were low-pass filtered (6 and 45 Hz, respectively) with a dual-pass, 2nd order Butterworth filter.

Temporal-spatial parameters, knee joint kinematics, and knee joint kinetics were calculated in Visual3D (C-Motion, Germantown, MD, USA). Briefly, bilateral gait events (i.e., heel strike and toe off) were first determined using a foot kinematic-based detection algorithm^23^; temporal-spatial outcomes were derived (walking speed, stride length, stride width, and cadence). Peak sagittal knee angles from each gait cycle were extracted to compute joint range of motion (ROM). Joint angles were computed using a flexion–extension, ab/adduction, axial rotation sequence. Inverse dynamics were used to calculate external knee adduction moment (KAM) and knee flexion moment (KFM), resolved relative to the proximal (thigh) segment; peak KAM and KFM were extracted, as were KAM impulse (time integral of KAM) and KAM loading rate (slope between 20 and 80% of time from heel strike to first peak). Knee joint contact forces (total, medial, and lateral) were calculated based on the model of Schipplein and Andriacchi, and developed as described in previous literature^24,25^. All joint kinematic and kinetic parameters are only reported for the contralateral limb (i.e., non-limb loss).

### Physiological biomarkers

Fifteen SM provided serum samples at baseline and follow-up, while four (of eight at baseline) SM opted to have synovial fluid samples taken at follow-up. Whole blood samples were collected through venipuncture; serum was obtained through centrifugation at 1000RPM for 10 min at room temperature. Synovial fluid was aspirated from the contralateral knee by a trained physician. After collection, both biofluids were aliquoted and stored at − 80 °C for downstream analyses. Subsequently, serum and synovial fluid samples were analyzed for collagen II cleavage (C2C), N-Propeptide of Collagen IIA (PIIANP), hyaluronic acid (HA), and cartilage oligomeric matrix protein (COMP) as markers of osteochondral (bone and cartilage) remodeling; and C-Telopeptide of Type 1 Collagen (CTX-1) and N-telopeptide of Type 1 Collagen (NTX-1) as markers of subchondral bone degradation via Enzyme-Linked Immunosorbent Assays according to manufacturer’s protocol. Serum and synovial fluid samples were also analyzed for various inflammatory and tissue-remodeling markers via multiplex bead assays. For markers related to collagen and mineral metabolism, custom multiplex kits (Invitrogen) were used to evaluate the following markers: matrix metalloprotease (MMP) -2, MMP3, MMP7, MMP8, MMP9, MMP12, MMP13, and tissue-inhibitor or metalloprotease (TIMP)-1. Specifically, for cytokines and chemokines, a ProcartaPlex Human Cytokine/Chemokine kit (Invitrogen, Carlsbad, CA, USA) was used according to manufacturer recommendations.

### Patient-reported outcomes

Patient-reported outcomes were obtained from all SM at baseline (n = 17) and follow-up (n = 17), including pain severity by Visual Analog Scale (VAS)^26^ and PROMIS Pain Interference (SF-8a), Knee Injury and Osteoarthritis Outcome Score (KOOS)^27^, and Veterans RAND 36-Item Health Survey (VR-36)^28^.

### Statistical analyses

Statistical analyses were performed using Statistical Package for Social Science (SPSS) software (version 25; Chicago, IL, USA). Sample characteristics are presented as numbers, percentages, means ± standard deviations. Normal distribution was evaluated using the Shapiro–Wilk tests. To first compare outcomes between timepoints (i.e., baseline vs. two-year follow-up), paired sample *t-*tests, or Wilcoxon signed-rank tests (where applicable), were used to compare all outcomes within each domain: (i) demographics, (ii) radioanatomy, (iii) gait biomechanics, (iv) physiological biomarkers, and (v) patient-reported. Effect sizes are reported using Cohen’s d. Chi-square analysis was utilized to compare the proportions of KL classifications. Second, associations between each outcome (at baseline) with changes in tibiofemoral and patellofemoral joint space (from baseline to two-year follow-up) were investigated using Pearson’s correlations. Kendall’s tau-b correlation was utilized for correlations between Insall-Salvati proportions and joint space narrowing. Values of *p* < 0.05 were considered significant.

## Results

### Clinical imaging

Upon follow-up (mean time elapsed since baseline evaluation = 26.6 ± 3.2 mo), knee joint health worsened, particularly among seven SMs (2 transtibial, 5 transfemoral) who presented at baseline with no clinical joint degeneration (KL = 0) within the contralateral knee; all returned at two-year follow-up with evidence of degeneration (KL ≥ 1). The proportion of SM with KL ≥ 2, a frequently used threshold for OA diagnoses, was not different at either time point (*p* = 0.28). Medial tibiofemoral joint space was similar (*p* = 0.46) between time points, but lateral tibiofemoral joint space decreased by 7.9% (*p* = 0.01) from baseline to follow-up (Table 1). There were no changes in medial (− 15.1%, *p* = 0.35) and lateral (− 18.2%, *p* = 0.20) patellofemoral joint spaces.

**Table 1:** Mean ± standard deviation participant demographics, joint space measurement, and knee joint health at baseline and 2-year follow-up.

	Baseline (n = 17)	Follow-up (n = 17)	*P*-value
Age (yr)	37.7 ± 6.6	39.7 ± 6.7	< 0.01*
Stature (m)	1.78 ± 0.05	1.78 ± 0.05	0.73
Mass (kg)	87.7 ± 17.8	88.7 ± 18.7	0.17
Level of limb loss	Transtibial: 9 Transfemoral: 8		
Residual limb length (cm)	21.5 ± 8.76		
Time since injury (month)	119.8 ± 89.3		
Time since surgery (month)	108.2 ± 89.3		
Time since first prosthesis (month)	106.2 ± 90.0		
Time between visits (month)	26.6 ± 3.2		
Medial joint space (mm)^a^	5.83 ± 1.01	5.59 ± 1.18	0.46
Lateral joint space (mm)^a^	6.59 ± 1.13	6.09 ± 0.74	0.01*
Medial patellofemoral joint space (mm)^b^	8.14 ± 2.57	7.00 ± 1.96	0.35
Lateral patellofemoral joint space (mm)^b^	7.93 ± 2.49	6.61 ± 1.66	0.20
Kellgren–Lawrence grade; n(%)^b^			
0	7 (58.3%)	–	< 0.01*
1	3 (25.0%)	7 (58.3%)	0.03*
2	1 (8.3%)	3 (25.0%)	0.22
3	1 (8.3%)	2 (16.7%)	0.51
4	–	–	–
% with KL ≥ 2	2 (16.7%)	5 (41.7%)	0.28
Outerbridge—Medial^c^			
0	4 (50.0%)	5 (62.5%)	
1	1 (12.5%)	1 (12.5%)	
2	–	–	
3	2 (25.0%)	1 (12.5%)	
4	1 (12.5%)	1 (12.5%)	
Outerbridge—Lateral^c^			
0	6 (75.0%)	4 (50.0%)	
1	1 (12.5%)	3 (37.5%)	
2	1 (12.5%)	–	
3	–	–	
4	–	1 (12.5%)	
Outerbridge—Patellofemoral^c^			
0	3 (37.5%)	1 (12.5%)	
1	1 (12.5%)	1 (12.5%)	
2	–	5 (62.5%)	
3	2 (25.0%)	–	
4	2 (25.0%)	1 (12.5%)	

^a^Baseline and follow-up measures (n = 13).

^b^Baseline and follow-up measures (n = 12).

^c^Baseline and follow-up measures (n = 8).

Bolded values with asterisks indicate significance (*p* ≤ 0.05).

At follow-up, there was a greater sulcus angle (Table 2; *p* = 0.01) and lesser Insall-Salvati measurement (*p* = 0.03); there were no differences in the proportion of patella alta (Insall-Salvati > 1.2; *p* = 0.28) or patella baja (Insall-Salvati < 0.8; *p* = 0.25). Patellar tilt angle at baseline was positively correlated with medial and lateral patellofemoral joint space narrowing; r[12] = 0.70, *p* = 0.01 and r[12] = 0.71, *p* = 0.01, respectively. Insall-Salvati at baseline was negatively correlated with narrowing of the medial and lateral tibiofemoral joint spaces; r[13] =  − 0.49; *p* = 0.02, and r[13] =  − 0.47, *p* = 0.04, respectively.

**Table 2:** Mean ± standard deviation radioanatomic outcomes at baseline and 2-year follow-up, as well as correlations with changes (Δ) in tibiofemoral and patellofemoral joint space.

	Baseline (n = 13)	Follow-up (n = 13)	*P*-value	Effect size (Cohen’s d)	Δ Medial Tibiofemoral Joint Space	Δ Lateral Tibiofemoral Joint Space	Δ Medial Patellofemoral Joint Space	Δ Lateral Patellofemoral Joint Space
Patellar tilt (°)	8.73 ± 5.82	8.43 ± 4.33	0.9	0.06	0.12	− 0.44	0.70*	0.71*
Sulcus angle (°)	122.58 ± 9.43	132.18 ± 8.27	0.01*	1.08	0.18	− 0.04	0.11	− 0.07
Bisect offset (%)	57.6 ± 5.4	58.8 ± 6.5	0.53	0.20	0.27	0.16	− 0.10	− 0.52
Femorotibial angle (°)	3.42 ± 3.06	4.30 ± 2.58	0.42	0.31	0.31	0.33	0.46	0.22
Knee rotation (°)^a^	3.57 ± 4.00	3.07 ± 7.04	0.82	0.09	− 0.26	− 0.03	− 0.37	− 0.11
Insall-Salvati	1.06 ± 0.19	1.01 ± 0.19	0.03*	0.26	− 0.49*	-0.47*	-0.17	0.22
Alta (> 1.2)	5	2	0.28	–	–	–	–	–
Baja (< 0.8)	1	3	0.25	–	–	–	–	–

Bolded values with asterisks indicate significance (*p* ≤ 0.05).

### Gait biomechanics

At follow-up, there was lesser sagittal knee ROM (Table 3; *p* = 0.03) and decreased knee flexion moment (*p* = 0.05). Peak KAM at baseline was negatively associated with lateral patellofemoral joint space narrowing (r[12] =  − 0.58, *p* = 0.05), and positively associated with lateral tibiofemoral joint space narrowing (r[13] = 0.64, *p* = 0.02). KAM loading rate at baseline was negatively associated with medial patellofemoral joint space narrowing (r[12] =  − 0.80, *p* = 0.002) and lateral patellofemoral joint space narrowing (r[12] =  − 0.75, *p* = 0.005). KAM impulse at baseline was positively correlated with medial tibiofemoral joint space narrowing (r[13] = 0.61, *p* = 0.61). Peak medial joint contact forces at baseline were negatively associated with lateral patellofemoral joint space narrowing (r[12] =  − 0.66, *p* = 0.02). Peak lateral joint contact forces at baseline were negatively associated with medial tibiofemoral joint space narrowing (r[13] =  − 0.64, *p* = 0.02).

**Table 3:** Mean ± standard deviation gait biomechanical outcomes at baseline and 2-year follow-up, as well as correlations with changes (Δ) in tibiofemoral and patellofemoral joint space.

	Baseline (n = 17)	Follow-up (n = 17)	*P*-value	Effect Size (Cohen’s d)	Δ Medial Tibiofemoral Joint Space	Δ Lateral Tibiofemoral Joint Space	Δ Medial Patellofemoral Joint Space	Δ Lateral Patellofemoral Joint Space
Walking speed (m/s)	1.32 ± 0.12	1.30 ± 0.10	0.61	0.18	− 0.16	0.12	− 0.24	0.14
Stride length (m)	1.48 ± 0.09	1.47 ± 0.08	0.90	0.12	− 0.41	0.19	− 0.31	0.08
Stride width (m)	0.17 ± 0.03	0.17 ± 0.03	0.82	0.00	0.31	0.20	0.44	0.19
Cadence (steps/min)	107.1 ± 6.0	106.1 ± 5.7	0.31	0.17	− 0.28	− 0.36	− 0.29	− 0.08
Peak knee flexion (°)	68.1 ± 5.9	69.3 ± 6.2	0.41	0.20	0.12	− 0.31	0.30	0.24
Sagittal knee range of motion (°)	69.3 ± 6.2	66.6 ± 4.2	0.03*	0.51	− 0.06	− 0.13	0.01	− 0.13
Knee adduction moment (KAM) peak (BW·Ht)	1.64 ± 0.72	1.76 ± 0.88	0.49	0.15	0.41	0.64*	− 0.29	− 0.58*
KAM loading rate (BW·Ht·s^−1^)	4.48 ± 1.25	4.93 ± 1.74	0.32	0.30	0.05	0.42	− **0.80**	− 0.75*
KAM impulse (BW·Ht·s)	0.72 ± 0.37	0.79 ± 0.37	0.23	0.19	0.61*	0.48	− 0.06	− 0.51
Knee flexion moment peak (BW·Ht)	8.17 ± 3.36	6.56 ± 3.08	0.05*	0.50	− 0.39	− 0.42	0.05	0.43
Joint contact forces (BW)								
Total	3.60 ± 0.41	3.71 ± 0.43	0.45	0.26	− 0.16	0.05	0.08	0.19
Medial	2.75 ± 0.41	2.84 ± 0.39	0.52	0.22	0.11	0.53	− 0.53	− 0.66*
Lateral	1.45 ± 0.26	1.40 ± 0.29	0.56	0.18	− 0.64*	− 0.38	− 0.19	0.30

Correlations in bold indicate *p* < 0.05. Correlation in bold and italicized indicate *p* < 0.01.

Bolded values with asterisks indicate significance (*p* ≤ 0.05).

### Physiological biomarkers

At follow-up, there were greater serum concentrations of CTX-1 (+ 87.0%; *p* < 0.001), HA (+ 80.4%; *p* < 0.001), COMP (+ 148.0%; *p* < 0.001; d = 1.29), SDF-1 (+ 57.9; *p* = 0.002), MMP-12 (+ 203.0%; *p* < 0.001), MMP-13 (+ 70.4%; *p* = 0.04), MMP-7 (+ 40.8%; p = 0.03), and MMP-8 (+ 156.0%, p = 0.03; Table 4); in contrast, there were lesser concentrations of C2C (− 28.9%; *p* = 0.01), PIIANP (− 504.1%; *p* = 0.001), NTX-1 (− 67.8%; *p* = 0.02), CCL-4 (− 47.7%; *p* < 0.001), CCL-11 (− 52.2%; *p* = 0.001), CXCL-10 (− 39.7; *p* = 0.001), IFN-α(− 60.1%; *p* = 0.004), and IL-1α (− 85.1%; *p* < 0.001). Concentrations of CCL-2 and IL-18 at baseline were negatively associated with lateral tibiofemoral joint space narrowing (r[12] = − 0.60, *p* = 0.04 and r[12] = − 0.74, *p* = 0.01, respectively). Concentrations of CTX-1 at baseline were positively associated with lateral patellofemoral joint space narrowing (r[11] = − 0.83, *p* = 0.001).

**Table 4:** Mean ± standard deviation blood serum biomarkers at baseline and 2-year follow-up, as well as correlations with changes in tibiofemoral and patellofemoral joint space.

	Baseline (n = 14)	Follow-up (n = 14)	*P*-value	Effect size (Cohen’s d)	Δ Medial Tibiofemoral joint space	Δ Lateral Tibiofemoral joint space	Δ Medial Patellofemoral joint space	Δ Lateral Patellofemoral joint space
CTX-1 (ng/ml)	0.23 ± 0.12	0.43 ± 0.15	< 0.001*	1.47	− 0.23	− 0.15	0.55	0.83*
HA (ng/ml)	68.78 ± 88.51	124.07 ± 86.52	< 0.001*	0.63	− 0.28	− 0.32	0.23	0.26
C2C (ng/ml)	433.84 ± 159.92	308.45 ± 65.63	0.01*	1.03	− 0.04	0.41	0.31	0.31
TIMP-1 (pg/ml)	338,319.00 ± 749,252.29	68,259.00 ± 104,356.49	0.21	0.50	− 0.18	− 0.05	0.23	0.26
MMP-2 (pg/ml)	424.00 ± 660.51	579.02 ± 504.20	0.08	0.26	0.20	− 0.21	0.03	− 0.26
MMP-3 (pg/ml)	720.73 ± 579.23	581.78 ± 311.36	0.34	0.30	− 0.14	− 0.24	0.17	0.15
MMP-9 (pg/ml)	128.85 ± 111.36	262.35 ± 434.64	0.19	0.42	− 0.28	− 0.34	0.08	0.33
MMP-12 (pg/ml)	12.17 ± 8.29	36.87 ± 22.03	< 0.001*	1.48	− 0.17	− 0.51	− 0.30	− 0.28
MMP-13 (pg/ml)	13.57 ± 25.18	23.13 ± 11.81	0.04*	0.49	0.17	− 0.16	0.25	0.21
MMP-7 (pg/ml)	409.94 ± 343.29	577.20 ± 458.83	0.03*	0.41	0.32	− 0.10	− 0.11	− 0.32
MMP-8 (pg/ml)	22.10 ± 13.87	56.58 ± 55.80	0.03*	0.85	− 0.22	− 0.34	− 0.04	0.11
NTX-1 (ng/ml)	24.86 ± 22.25	8.01 ± 6.49	0.02*	1.47	0.40	0.08	− 0.03	− 0.26
CCL-2 (pg/ml)	26.79 ± 26.93	25.90 ± 21.36	0.82	0.63	− 0.48	− 0.60*	− 0.26	− 0.03
CCL-4 (pg/ml)	102.74 ± 40.25	53.77 ± 16.67	< 0.001*	1.03	− 0.22	0.05	0.06	0.07
CCL-5 (pg/ml)	8.44 ± 4.55	8.60 ± 4.23	0.91	0.50	0.25	0.10	− 0.58	− 0.36
CCL-11 (pg/ml)	56.79 ± 40.78	27.14 ± 22.73	0.001*	0.26	− 0.21	− 0.27	0.08	0.38
CXCL-10 (pg/ml)	14.26 ± 7.15	8.60 ± 2.92	0.001*	0.30	− 0.31	0.12	− 0.29	− 0.14
COMP (pg/ml)	28,267.94 ± 22,701.9	70,112.62 ± 39,752.34	< 0.001*	0.42	0.27	0.05	0.12	− 0.13
INF-a (pg/ml)	1.73 ± 1.02	0.69 ± 0.35	0.004*	1.48	0.18	− 0.28	0.32	0.03
IL-18 (pg/ml0	16.63 ± 16.39	19.21 ± 14.06	0.427	0.49	− 0.41	− 0.74*	− 0.18	0.08
IL-1a (pg/ml)	4.63 ± 1.30	0.69 ± 0.35	< 0.001*	0.41	0.04	0.36	− 0.15	− 0.24
IL-7 (pg/ml)	1.83 ± 2.93	0.73 ± 0.44	0.15	0.85	− 0.23	− 0.40	0.25	0.37
SDF-1 (pg/ml)	281.93 ± 121.22	445.20 ± 96.44	0.002*	1.49	0.13	0.18	− 0.12	− 0.26

Bolded values with asterisks indicate significance (*p* ≤ 0.05).

At follow-up, there were greater synovial fluid concentrations of CCL-11 (+ 133.3%, *p* = 0.05; Table 5). Moreover, concentrations of MMP-13 at baseline were positively associated with medial tibiofemoral joint space narrowing (r[4] = 0.99, *p* = 0.001). Concentrations of MMP-8 at baseline were positively associated with lateral patellofemoral joint space narrowing (r[3] = 0.99, p = 0.01). Concentrations of MMP-1 at baseline were negatively associated with lateral patellofemoral joint space narrowing (r[3] = − 0.99, *p* = 0.04).

**Table 5:** Mean ± standard deviation knee joint synovial fluid biomarkers at baseline and 2-year follow-up, as well as correlations with changes (Δ) in tibiofemoral and patellofemoral joint space.

	Baseline (n = 8)	Follow-up (n = 4)	*P*-value	Effect Size (Cohen’s d)	Δ Medial Tibiofemoral Joint Space	Δ Lateral Tibiofemoral Joint Space	Δ Medial Patellofemoral Joint Space	Δ Lateral Patellofemoral Joint Space
C2C (ng/ml)	0.11 ± 0.07	0.06 ± 0.06	0.45	0.74	0.88	0.44	− 0.25	0.03
PIIANP (ng/ml)	3.21 ± 5.13	2.45 ± 4.00	0.85	0.16	0.83	0.38	0.76	0.91
NTX-1 (ng/ml)	0.02 ± 0.01	0.01 ± 0.00	0.35	1.20	0.94	0.72	− 0.38	− 0.62
CCL-2 (pg/ml)	2.28 ± 1.88	1.07 ± 0.41	0.31	0.76	0.47	0.52	− 0.94	− 0.80
CXCL-10 (pg/ml)	0.16 ± 0.12	0.12 ± 0.01	0.58	0.40	0.56	0.35	0.48	0.21
CCL-11 (pg/ml)	0.03 ± 0.03	0.07 ± 0.02	0.05*	1.46	0.42	0.02	0.8	0.60
IL-18 (pg/ml)	0.65 ± 0.66	0.86 ± 0.18	0.56	0.37	0.18	− 0.04	0.92	0.78
IL-7 (pg/ml)	0.04 ± 0.03	0.02 ± 0.01	0.31	0.78	0.33	− 0.25	0.71	0.88
SDF-1 (pg/ml)	9.05 ± 6.76	7.62 ± 5.22	0.73	0.23	0.47	0.39	− 0.97	− 0.87
MMP-12 (pg/ml)	0.06 ± 0.03	0.05 ± 0.10	0.81	0.17	0.95	0.88	0.02	− 0.26
MMP-13 (pg/ml)	0.40 ± 0.15	0.30 ± 0.21	0.10	0.59	0.99*	0.81	− 0.81	− 0.94
MMP-7 (pg/ml)	0.15 ± 0.13	0.17 ± 0.11	0.25	0.16	0.67	0.67	0.40	0.13
MMP-8 (pg/ml)	0.18 ± 0.20	0.56 ± 0.72	0.38	0.90	0.56	0.70	0.96	0.99*
MMP-1 (pg/ml)	12.09 ± 19.80	4.95 ± 5.48	0.50	0.42	0.82	0.36	− 0.94	− 0.99*

Bolded values with asterisks indicate significance (*p* ≤ 0.05).

### Patient-reported outcomes

At follow-up, SM did not report greater pain severity within the contralateral leg (*p* = 0.34), ipsilateral leg (*p* = 0.13), contralateral knee (*p* = 0.27), ipsilateral knee (for transtibial; *p* = 0.10), back (*p* = 0.13), or whole body (*p* = 0.54; Table 6). SM reported decreasing values in KOOS subdomains of Symptoms (*p* = 0.03) and Quality of Life (*p* = 0.01) from baseline to follow-up, suggesting worsening symptoms and diminishing quality of life. SM also reported decreasing values in the VR-36 subdomain of General Health (*p* = 0.03) from baseline to follow-up, again suggesting diminishing general health. There were no differences in PROMIS (all *p* > 0.05). Changes in medial tibiofemoral joint space were negatively correlated with baseline KOOS-Sport (r[16] = − 0.69; *p* = 0.01) and VR-36 General Health (r[16] = − 0.69, *p* = 0.01).

**Table 6:** Mean ± standard deviation patient-reported outcomes at baseline and 2-year follow-up, as well as correlations with changes (Δ) in tibiofemoral and patellofemoral joint space.

	Baseline (n = 17)	Follow-up (n = 17)	*P*-value	Effect Size (Cohen’s d)	Δ Medial Tibiofemoral Joint Space	Δ Lateral Tibiofemoral Joint Space	Δ Medial Patellofemoral Joint Space	Δ Lateral Patellofemoral Joint Space
Pain severity (visual analog scale [VAS]; mm)								
Intact Leg	8.46 ± 13.43	12.64 ± 15.55	0.34	0.29	− 0.09	− 0.24	− 0.11	0.07
Residual Leg	10.29 ± 13.29	17.88 ± 22.19	0.13	0.41	0.21	0.15	− 0.09	− 0.19
Intact Knee	8.62 ± 11.26	12.25 ± 16.90	0.27	0.25	0.33	− 0.10	0.04	0.03
Residual Knee	4.47 ± 10.54	15.85 ± 24.52	0.10	0.60	− 0.24	0.18	− 0.09	0.01
Whole Body	14.28 ± 16.26	16.84 ± 15.83	0.54	0.16	− 0.03	− 0.31	0.02	0.19
Back	17.38 ± 14.83	27.87 ± 27.27	0.13	0.48	0.04	− 0.54	0.37	0.53
Knee injury and osteoarthritis outcome score (KOOS)								
Pain	86.41 ± 11.65	79.00 ± 18.73	0.05	0.48	− 0.52	0.02	− 0.07	0.06
Symptoms	88.71 ± 8.62	81.65 ± 16.61	0.03*	0.53	− 0.45	0.02	0.05	0.22
Activities of Daily Living	90.47 ± 11.66	85.76 ± 18.92	0.18	0.30	− 0.49	− 0.08	− 0.11	− 0.04
Sport	79.41 ± 19.91	73.53 ± 23.17	0.27	0.27	− 0.69*	− 0.09	− 0.33	0.12
Quality of Life	74.59 ± 21.05	61.65 ± 27.15	0.01*	0.53	− 0.46	0.02	0.01	0.25
VR-36								
Physical Function	66.25 ± 29.58	61.25 ± 26.24	0.5	0.18	− 0.57	− 0.21	− 0.02	0.14
Bodily Pain	61.69 ± 23.45	65.94 ± 20.63	0.22	0.19	− 0.03	0.08	0.07	− 0.17
General Health	69.06 ± 26.09	56.50 ± 16.35	0.03*	0.58	− 0.69*	− 0.38	− 0.25	− 0.10
Vitality	55.94 ± 27.70	57.50 ± 28.93	0.74	0.06	− 0.48	− 0.18	0.03	0.09
Social Functioning	76.56 ± 26.17	69.21 ± 25.82	0.29	0.28	− 0.37	− 0.29	0.17	0.12
Mental Health	79.75 ± 16.16	75.00 ± 18.04	0.19	0.28	− 0.43	− 0.43	0.00	0.14
Role Limitation due to Emotional Problems	76.04 ± 32.61	80.75 ± 21.89	0.43	0.17	− 0.24	− 0.14	0.27	0.17
Role Limitation due to Bodily Problems	70.75 ± 44.58	68.36 ± 39.70	0.79	0.06	− 0.34	− 0.13	0.09	0.07
Physical Score	43.10 ± 12.63	41.05 ± 9.52	0.41	0.18	− 0.46	− 0.13	− 0.05	− 0.03
Mental Score	51.80 ± 8.26	51.28 ± 9.71	0.76	0.06	− 0.37	− 0.35	0.17	0.18
PROMIS (SF-8A)	51.92 ± 8.81	52.32 ± 9.32	0.79	0.04	0.32	0.12	− 0.06	− 0.08

Bolded values with asterisks indicate significance (*p* ≤ 0.05).

## Discussion

This comprehensive, longitudinal evaluation for knee joint health in SM with unilateral lower limb loss aimed to identify relationships among associative risk factors at baseline with knee joint space narrowing. The primary hypotheses that decreases in medial tibiofemoral and patellofemoral joint spaces will be associated with increased knee joint kinetics and elevated serum concentrations, and synovial fluid inflammatory biomarkers of bone and cartilage metabolites, were partially supported.

Radiographically, KL grading suggests overall minimal to moderate joint degeneration (i.e., KL ≤ 3), with worsening of knee joint health from baseline to two-year follow-up (17–42% with KL ≥ 2). Of the seven SM (2 TT, 5 TF) who presented at baseline with no clinical joint degeneration (KL = 0), all returned at two-year follow-up with evidence of degeneration (KL ≥ 1) within the contralateral knee. Although ultimately excluded at follow-up, one participant (42 yrs of age) received a medial knee arthroplasty during the study period (KL = 3 at baseline, with evidence of degeneration in the medial [Outerbridge Grade = 4], lateral [Outerbridge Grade = 2], patellofemoral [Outerbridge Grade = 3] compartments). A secondary hypothesis predicted that radioanatomic measures (e.g., bisect offset, patellar tilt, and tibiofemoral joint angle) would be associated with more significant medial patellofemoral joint space narrowing; this hypothesis was partially supported, as patellar tilt was positively associated with medial and lateral patellofemoral joint space narrowing, consistent with prior literature in persons with patellofemoral pain and OA progression^18,29^. Furthermore, decreases in the Insall-Salvati ratio, a patellar height measurement, were negatively associated with medial and lateral tibiofemoral joint space narrowing. While the ratio of participants with patella alta/baja was similar between time points, changes in the Insall-Salvati ratio can impact patellofemoral joint mechanisms and often result in diminished function and deterioration of knee joint health^30^. Changes in patellar tendon length has been extensively studied following anterior cruciate ligament repairs and total knee arthroplasty, but not in the population of SM with limb loss, who present a unique morphological change, absent surgical intervention^31^. Our results concur with the literature, noting that longer patellar tendons (larger Insall-Salvati ratio) present a situation wherein the patella may be unstable and maltrack, impacting the articular surfaces of the knee and patella^32^. Within our study sample, decreases in the patellar height could suggest a more proximal patellar positioning, prior to study enrollment. Some potential explanations for a decreased Insall-Salvati ratio may be due to increases in weight or forces upon the contralateral knee, or decreases in the retropatellar fat pad^33–35^. Although literature notes a higher prevalence of patella baja with greater body mass^33^, it can be surmised that the increased dependence on the contralateral limb after unilateral limb loss may mimic similar overloading conditions of overweight/obesity. Further research is needed to longitudinally track morphological changes in knee radioanatomy and patellar anatomy of the contralateral knee immediately following limb loss, as our study population at enrollment were several years past their injury.

Biomechanically, while cross-sectional evaluations indicate larger knee joint loads in persons with vs. without lower limb loss, here there was a general lack of changes between timepoints in the current study. Common biomechanical adaptations during walking with vs. without knee OA, though variable across studies^36^, include lesser knee flexion–extension excursion (largely due to joint stiffness or pain) or greater loading metrics (e.g., external KAM peak or loading rate). While prior studies have identified differences in joint loads with vs. without knee OA, such changes are more apparent with more severe stages of OA (i.e., KL ≥ 3)^37^, and peak KAM is positively correlated with the severity of knee OA (medial tibiofemoral joint space narrowing)^38,39^. Here, despite the similar (peak) joint loads between timepoints, and minimal-moderate joint degeneration (i.e., KL1-3), there were several moderate associations between KAM loading measures and tibiofemoral or patellofemoral joint space narrowing. We and others have suggested that idiopathic knee OA after limb loss results not necessarily from chronically high joint loads but rather from “unusual” joint loads, defined as loads the joint is not conditioned to sustain frequently^24,40,41^. Exposure to such unusual loads could be particularly hazardous to long-term joint health if introduced following an extended period of low activity and ostensible structural weakening, such as if an individual with unilateral limb loss hops on one leg (e.g.,^42^). However, more ecologically valid methods are needed to comprehensively track biomechanical and mobility outcomes for prolonged durations and across numerous activities, at home and in the community (vs. exclusively in a lab during controlled conditions).

Physiologically, while numerous serum biomarkers changed from baseline to follow-up, CTX-1 specifically was positively correlated with lateral patellofemoral joint space narrowing and highlights the presence of increased osteochondral breakdown in systemic biomarkers (i.e., serum). However, we must acknowledge that SM with limb loss often present with multi-site musculoskeletal pain^43,44^ which may confound joint-specific musculoskeletal research, particularly in the interpretation of some systemic biomarkers and other outcomes (i.e., gait biomechanics, patient-reported function or quality of life). In the current sample, while participants reported multi-site pain (e.g., residual limb, low back), these did not change from baseline to two-year follow-up. Nevertheless, more specific to the knee joint, we identified increases in MMP-13 and MMP-8 among synovial fluid at follow-up, which were respectively associated with greater narrowing of the medial tibiofemoral and lateral patellofemoral joint spaces. These MMP biomarkers indicate joint catabolic activity, ultimately leading to the loss of cartilage via inhibition of cartilage differentiation and promotion of chondrocyte apoptosis^45,46^. This is a significant finding and novel contribution to both the limb loss and non-limb loss OA populations.

Patient-reported outcomes provide important insights on symptom severity and corresponding influences on well-being and quality of life; however, the ability to accurately assess pain severity is likely hampered in the SM population. For example, while pain experience can be subjective and thus difficult to compare between participants or populations, SM tend to under-report symptoms in both clinical and research settings^47^. Outcome measures such as the KOOS or VR-36 may be more appropriate to understand the impact and monitor changes in knee joint health among SM with limb loss. Here, KOOS scores generally indicated better outcomes (i.e., scores closer to 100) than reported in the literature across a range of OA pathologies^48^, for most subdomains. Also, only Symptoms and Quality of Life decreased from baseline to two-year follow-up, again noting a relatively lesser severity of degeneration within the current sample. VR-36 and SF-8A outcomes of the current sample provide a slightly different perspective from acute pain alone, with decreasing General Health from baseline to two-year follow-up, but similarly suggest overall better general health and lesser pain interference in comparison to existing literature^49,50^.

## Limitations

Several limitations are present and should be considered. Acknowledging a general paucity of longitudinal data, many patients with knee OA enrolled in longitudinal studies do not progress radiographically during ~ two-year trials, highlighting that OA progression is highly variable by individual and stage of disease^9,51,52^; in the absence of existing characterization of disease trajectory among SM with limb loss, we ultimately chose a two-year follow-up window to balance additional pragmatic considerations (i.e., attrition). Moreover, our broad cross-sectional recruitment strategy (i.e., no a priori grouping by joint health or other factors upon enrollment) generated a convenience sample with somewhat heterogeneous characteristics. Despite the likelihood for knee joint health to be influenced by time since injury and/or “severity” of injury (i.e., transtibial vs. transfemoral limb loss), neither the study design nor final dataset support the evaluation of such factors within the longitudinal framework. The prevalence of new amputations within the military has decreased every year since peaking in 2011^53^; while our sample is more reflective of SM injured within this time period (time since injury = 119.8 ± 89.3 months), without baseline measures obtained more proximal (and/or prior) to time of injury we are unable to determine the causality of observed associations. While attrition is an inherent challenge for longitudinal studies, this challenge was magnified during the COVID-19 global pandemic as travel and research restrictions prevented many participants from returning for follow-up. Importantly, compared to participants who completed follow-up, participants lost to follow-up were not different by baseline demographics, medical history, or knee joint health grading (Supplementary Table 1); thus, mitigating potential confounding effects of attrition beyond sample size alone.

## Conclusion

This study establishes a comprehensive approach for evaluating (contralateral) knee joint health after unilateral lower limb loss and emphasizes the need for early identification/intervention to mitigate the fast-progressing degeneration among SM with lower limb loss. In particular, SM with traumatic limb loss are typically younger at time of injury and more active following limb loss, increasing the likelihood of suffering long-term consequences of poor knee joint health on function and quality of life. Future research should continue to employ this multi-faceted approach to evaluate knee joint health and encourage the development of optimal interventions to maximize quality of life and long-term functional outcomes for SM with limb loss.

### Supplementary Information

Supplementary Table S1.

## Data Availability

All data associated with this study are present in the paper. Availability of raw clinical imaging, biomechanical data, or serum samples are not available for public use.

## Abbreviations

C2C: Collagen II cleavage
COMP: Cartilage oligomeric matrix protein
CTX-1: C-Telopeptide of type 1 collagen
HA: Hyaluronic acid
KAM: Knee adduction moment
KFM: Knee flexion moment
KL: Kellgren–Lawrence
KOOS: Knee injury and osteoarthritis outcomes score
MMP: Matrix metalloprotease
MRI: Magnetic resonance images
NTX-1: N-telopeptide of type 1 collagen
PIIANP: N-propeptide of collagen IIA
OA: Osteoarthritis
OC: Outerbridge classification
SM: Service members
TIMP: Tissue-inhibitor or matelloprotease
VAS: Visual analog scale
VR36: Veterans RAND 36 item health survey
