# Supplementary material for: Exploring relationships among multi-disciplinary assessments for knee joint health in service members with traumatic unilateral lower limb loss: a two-year longitudinal investigation
Source: Sci Rep. 2023 Dec 1;13:21177. doi: 10.1038/s41598-023-48662-9 (PMC10692131; doi:10.1038/s41598-023-48662-9)
Supplement: Supplementary file 1 — Supplementary Table S1. [file 41598_2023_48662_MOESM1_ESM.docx]

| **Supplemental Table 1.** Demographics, knee joint health, and joint space measurements at baseline between participants who completed the study and those lost to follow-up. Values are presented as mean±standard deviation. | | | |
| --- | --- | --- | --- |
|  | **Completed Follow-Up (n=17)** | **Lost To Follow-Up (n=20)** | **p (sig)** |
| Age (yr) | 37.7±6.6 | 35.0±7.9 | 0.27 |
| Stature (m) | 1.78±0.05 | 1.81±0.06 | 0.13 |
| Mass (kg) | 87.7±17.8 | 93.6±12.9 | 0.25 |
| Level of Limb Loss | Transtibial: 9  Transfemoral: 8 | Transtibial: 14  Transfemoral: 6 | 0.29 |
| Residual Limb Length (cm) | 21.5±8.76 | 22.3±7.9 | 0.79 |
| Time Since Injury (month) | 119.8±89.3 | 107.5±52.8 | 0.61 |
| Time Since Surgery (month) | 108.2±89.3 | 92.8±55.3 | 0.53 |
| Time Since First Prosthesis (month) | 106.2±90.0 | 90.7±55.3 | 0.53 |
| Medial Joint Space (mm)^a^ | 5.83±1.01 | 5.87±1.07 | 0.92 |
| Lateral Joint Space (mm) ^a^ | 6.59±1.13 | 6.63±0.87 | 0.92 |
| Medial Patellofemoral Joint Space (mm)^b^ | 8.14±2.57 | 7.23±2.42 | 0.35 |
| Lateral Patellofemoral Joint Space (mm)^b^ | 7.93±2.49 | 7.69±3.28 | 0.83 |
|  |  |  |  |
| Kellgren-Lawrence Grade; n(%)^c^ |  |  |  |
| 0 | 7 (58.3%) | 10 (62.5%) | 0.81 |
| 1 | 3 (25.0%) | 4 (25.0%) | 0.74 |
| 2 | 1 (8.3%) | 1 (6.3%) | 0.96 |
| 3 | 1 (8.3%) | 1 (6.3%) | 0.96 |
| 4 | - | - |  |
| % with KL≥2 | 2 (16.7%) | 2 (12.5%) | 0.95 |
| Outerbridge – Medial^d^ |  |  |  |
| 0 | 4 (50.0%) | 8 (72.7%) | 0.31 |
| 1 | 1 (12.5%) | - | 0.23 |
| 2 | - | - |  |
| 3 | 2 (25.0%) | 2 (18.2%) | 0.72 |
| 4 | 1 (12.5%) | 1 (9.1%) | 0.81 |
| Outerbridge – Lateral^d^ |  |  |  |
| 0 | 6 (75.0%) | 9 (81.8%) | 0.72 |
| 1 | 1 (12.5%) | 1 (9.1%) | 0.81 |
| 2 | 1 (12.5%) | - | 0.23 |
| 3 | - | 1 (9.1%) | 0.38 |
| 4 | - | - |  |
| Outerbridge – Patellofemoral^d^ |  |  |  |
| 0 | 3 (37.5%) | 2 (18.2%) | 0.35 |
| 1 | 1 (12.5%) | 2 (18.2%) | 0.74 |
| 2 | - | - |  |
| 3 | 2 (25.0%) | 1 (9.1%) | 0.35 |
| 4 | 2 (25.0%) | 6 (54.5%) | 0.20 |
| a- Completed Follow-Up (n=16); Lost to Follow-Up (n=16) | |  |  |
| b- Completed Follow-Up (n=14); Lost to Follow-Up (n=13) | |  |  |
| c- Completed Follow-Up (n=12); Lost to Follow-Up (n=16) | |  |  |
| d- Completed Follow-Up (n=8); Lost to Follow-Up (n=11) | |  |  |
